# Factors affecting seafarers’ fatigue: a scoping review

**DOI:** 10.3389/fpubh.2025.1647685

**Published:** 2025-08-04

**Authors:** Mingfei Ma, Ruolin Liao

**Affiliations:** Department of International Law, Law School, Dalian Maritime University, Dalian, Liaoning, China

**Keywords:** seafarers, fatigue, maritime, shipping, factors

## Abstract

**Background:**

Nowadays, many maritime accidents occur due to the fatigue of seafarers. With the rapid development of the economy and society, the factors affecting seafarers’ fatigue are also changing. At present, there is a lack of systematic articles that examine the factors influencing seafarers’ fatigue over the past decade. This review aims to explore the various factors related to seafarers’ fatigue through a scoping review, to identify effective approaches to addressing the fatigue issues faced by seafarers.

**Methods:**

Studies were searched on PubMed, Science Direct. Academic search complete using EBSCOhost databases, Springer Nature Link, and Web of Science in May 2025. This scoping review was conducted based on the framework of Arksey and O’Malley and the Preferred Reporting items for Scoping Reviews flow diagram. The inclusion criteria were studies that determined the relationship between factors relevant to seafarers’ fatigue, including physical conditions and mental issues, etc. Data were narratively summarized and reported.

**Results:**

Eighteen articles were included in this review, while 18 major findings were clarified. Firstly, fatigue among seafarers has been frequently discussed over the past decade. Secondly, the factors influencing seafarers’ fatigue can be categorized into three aspects: the seafarer’s own factors, the working environment factors, and the management factors. The seafarer’s own factors include physiological factors, psychological factors, and sociological factors. The working environment factors include safety climate, external support, work demands, work mode, and ship conditions. The management factors include those at the ship, system, technology, industry, and national regulations levels.

**Conclusion:**

At the individual level, focus on the accumulation of psychological capital and enhancing work autonomy can alleviate fatigue. More solutions need to be implemented at the organizational level, including providing a better sleep environment, providing more external support, flexibly handling work demands and work patterns, and improving technology and management measures.

## 1 Introduction

It is of great significance to study the factors influencing seafarers’ fatigue, as it directly relates to maritime safety, operational efficiency, and the wellbeing of seafarers. Seafarers’ fatigue is one of the main causes of maritime accidents due to human factors. Understanding the factors influencing it can help formulate more effective risk management strategies and intervention measures.

The Maritime Safety Committee of the International Maritime Organization (IMO) approved the revised “Fatigue Guidelines” (MSC 1598) at its 100th meeting. The core of these guidelines also includes the view that since fatigue affects the safe operation of ships, fatigue management should naturally become an important part of the safety management system.

An extensive study published in 2006 by the Centre for Occupational and Health Psychology in Cardiff concluded that the potential for fatigue at sea is high due to seafarers’ exposure to many recognizable risk factors, operational, organizational, and environmental (1).

Fatigue is defined by the IMO as “a reduction in physical and/or mental capability as the result of physical, mental or emotional exertion which may impair nearly all physical abilities including strength; speed; reaction time; coordination; decision making; or balance” in Guidelines on Fatigue. A broader definition of fatigue is “the subjective experience of someone who is obliged to continue working beyond the point at which they feel confident of performing a task efficiently” (2). Researchers described it as an extremely disabling symptom both in healthy people and in various diseases (3).

Summed up, fatigue can be classified into physical and cognitive (mental) categories. Mental fatigue is believed to be psychological, whereas physical fatigue is considered synonymous with muscle fatigue (4, 5). Both physical and mental fatigue cause a decline in alertness, mental concentration, and motivation. Operating at a very high level of concentration, combined with a heavy workload over time, can result in high levels of mental fatigue. Consequently, the state of fatigue includes both a physical as well as a mental component, regardless of the nature of the preceding strain (i.e., physical or mental), as physiological and mental fatigue possibly have a common physiological basis. Extensive literature exists on fatigue within the medical, transportation, and psychology fields of research.

Acute fatigue is usually caused by excessive exertion in a short period, lack of sleep, mental stress, or infection, etc. (6, 7). It is a normal physiological response and usually recovers after rest (8). Short-term high-intensity activities, sleep deprivation, mental stress, and acute diseases (such as influenza or COVID-19) (6, 9, 70, 72) might cause acute fatigue. Chronic fatigue is a persistent state of exhaustion that cannot be alleviated even with adequate rest. Chronic fatigue may be related to various factors, including chronic diseases, psychological disorders, lifestyle and environmental factors (10, 11, 71).

The degree and dimensional character of fatigue depends on the form, dynamics and context of exertion. The context of exertion is described by the value and meaning of performance to the individual; rest and sleep history; circadian effects; psychosocial factors spanning work and home life (73); individual traits; diet; health, fitness and other individual states; and environmental conditions. The fatigue condition results in changes in strategies or resource use such that original levels of mental processing or physical activity are maintained or reduced (12).

Previous studies have mainly focused on the definition of seafarer fatigue as defined by the IMO. Some studies showed that a large number of seafarers suffered mental health problems and fatigue. For example, Hebbar and Mukesh surveyed 288 seafarers, of whom 40% felt unhappy, 30% endured stress and over 15% felt completely fatigued (13). Fatigue was reported to be associated with poor sleep. Similar findings were also reported by other researchers (14).

Also, there is another concept, digital fatigue, which is a state of mental, emotional and physical exhaustion caused by continuous and intense exposure to digital devices. This concept is associated with problems such as distraction, information overload and cognitive overload, especially caused by prolonged use of digital screens (15). The concept of digital fatigue has been studied, particularly in the education system, in relation to the negative effects of online learning on mental health (16). In this post-pandemic era filled with new technologies, it is possible that seafarers’ continuous work with digital systems may lead to similar fatigue.

An important fatigue factor is the safety climate, which reflects the attitudes, beliefs, perceptions, and values that persons share with safety at all levels of the organization (17). Safety climate is only one aspect of the safety culture in an organization. Safety culture can be defined as a constructed system of meaning through which the hazards of the world are understood (18). Safety culture is defined as a set of attitudes, perceptions, competencies, and patterns of behavior in an organization that encompasses “how people feel” about safety and safety management systems. The level of employee empowerment, higher management involvement and interest, the rewarding system, safety information, investment in safety, safety-oriented procedures, training, and the reporting system all reflect safety climate onboard ships (19).

Past literature reviews on seafarer fatigue or the study of fatigue in conjunction with other mental health issues (20) or the limitation of seafarer fatigue to a broader, similar psychological and physiological state (67) have lacked strict definitions.

During this study, it was learned that there are other similar symptoms such as breakdown and burnout. Given that the term “seafarer fatigue” corresponds to a specific physiological and psychological state and the necessity of conducting separate studies on the factors influencing seafarer fatigue, this paper only conducts a literature review on the factors influencing seafarer fatigue, attempting to clarify the clues and organize countermeasures, to better address seafarer fatigue, improve seafarers’ wellbeing, and reduce the occurrence of maritime accidents.

## 2 Methods

This scoping review was conducted based on the Arksey and O’Malley method framework (21) and the Preferred Reporting Items for Scoping Reviews flow diagram (PRISMA-ScR). The Arksey and O’Malley method framework contains five stages, which are identifying the research question, identifying relevant studies, study selection, charting the data, and collating, summarizing and reporting the results. As seafarers’ fatigue is a multi-factor and multi-dimensional issue (including physical, psychological, management, and environmental aspects), it requires a comprehensive exploration rather than focusing on a single intervention effect. Therefore, compared to systematic reviews that need to focus on the effect, a scoping review is more suitable for this study.

This review was guided by three review questions: “What fatigue symptoms are described in the literature for seafarers?” “Which factors affect these fatigue symptoms?” and “How do these factors affect the fatigue symptoms of seafarers?”

### 2.1 Searching strategy

Studies were searched using electronic databases, including PubMed, ScienceDirect, Academic Search Complete through EBSCOhost, Springer Nature Link, and Web of Science, to identify relevant published articles. Relevant research was searched for in May 2025. Studies were limited to peer-reviewed, written in English, and published from 2015 to 2025.

### 2.2 Eligibility criteria

The research questions guided the search terms, such as “seafarers fatigue,” and the inclusion criteria. Studies that met the inclusion criteria were eligible for review regardless of the age, gender, race and country of the participants. This study on seafarers is scheduled to follow Article II.1. (f) of the Maritime Labour Convention (MLC): “seafarer” means any person who is employed or engaged or works in any capacity on board a ship to which this Convention applies. And the definition of “ship” means a ship other than one that navigates exclusively in inland waters or waters within, or closely adjacent to, sheltered waters or areas where port regulations apply, according to Article II.1. (i) of MLC.

The included studies encompassed observational studies, qualitative studies, mixed-method studies, and experimental study designs conducted among workers in the maritime industry. However, reviews, letters, editorials, conference papers, policy statements, and books were excluded.

### 2.3 Study selection

**Figure 1:**
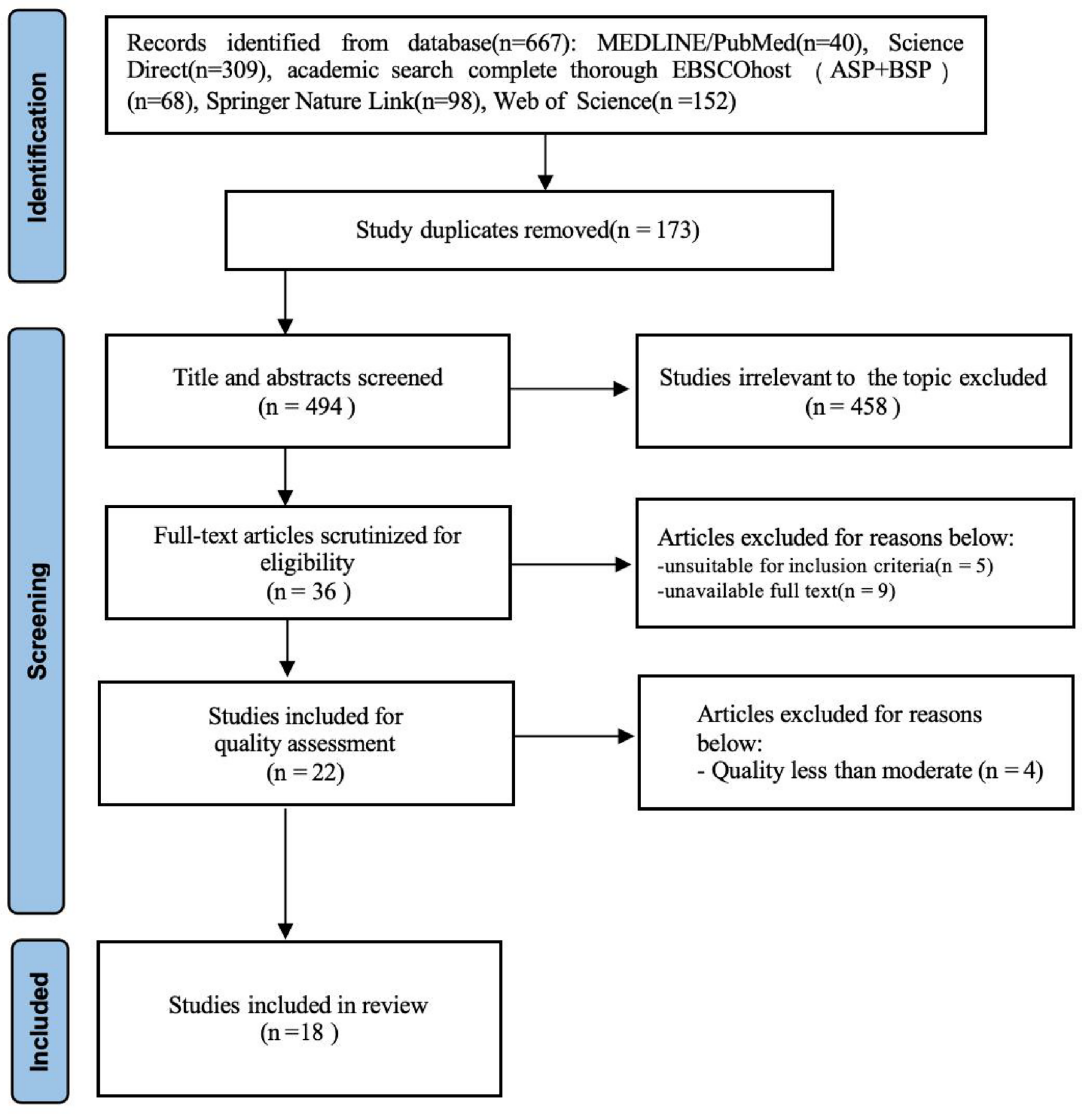
PRISMA-ScR flow diagram describing the process of study selection.

All identified studies were imported into EndNote 21. After removing duplicates, the predetermined inclusion criteria were applied, and a two-step process was used to evaluate these studies. First, a preliminary screening of the titles and abstracts was conducted, excluding those that were unrelated to the theme of this review; second, studies that met the inclusion and exclusion criteria were independently reviewed. Whenever there were differences among the researchers regarding the selection of the studies, the issues would be discussed until a consensus was reached with the research team. The PRISMA-ScR flowchart describing the process of study selection is shown in Figure 1.

### 2.4 Charting the data

Included studies were reviewed for characterizing general information: authors, publication year, country, study design, data collection, number of subjects, indicators, mental health problems, and related factors. They were charted into a Microsoft Excel database by the first author and verified by the other authors.

### 2.5 Collating, summarizing, and reporting the results

The characteristics charted in the Microsoft Excel database were narratively summarized. Type of study, mental health or psychological issues, statistically significant factors in quantitative studies, and all factors in qualitative studies were classified in domains for reporting and discussing.

## 3 Results

### 3.1 Searching and selecting the studies

A total of 667 studies were identified through the electronic databases. Then, 173 were excluded because of duplication. The titles and abstracts of 494 studies were screened to ensure they met the inclusion criteria. Next, 36 full texts were assessed for eligibility. Finally, 22 studies were selected for quality assessment. Four studies were excluded, as showing a quality lower than moderate. Consequently, 18 studies were included in the present review (Table 1).

**Table 1:** Characteristics of included studies.

Author (year)	Region (Country)	Study design	Data collection	Number of subjects	Indicator	Main finding
Zhao et al. (35)	Asia (China)	Mixed methods (quantitative study and qualitative study)	Quantitative questionnaire surveys and qualitative interviews	880 seafarers	Cardiff Seafarers’ Fatigue Research Program	Seafarers working for Chinese shipping companies had higher perceived fatigue levels than those working for European shipping companies. Various employment and on-board living and working conditions were associated with fatigue. In particular, lower job security, poorer environmental factors, higher job demands, increased workload and working hours, poorer sleep quality, and lack of rest, were all associated with fatigue.
Arıcan (30)	Asia (Turkey)	Cross-sectional study	Utilized sequential questionnaires, evaluated opinions in a non-adversarial manner, and provided repeated feedback on the current state of the group’s consensus.	150 seafarers	The Delphi Method and Multiple Regression Analysis	The high usage rate of digital screens has a significant impact on the daily activities of participants and may lead to an increase in their fatigue levels. The sleep mode is one of the most crucial factors influencing digital fatigue and overall mental health.
Pauksztat (24)	Europe (The United Kingdom)	Cross-sectional study	Collected from a convenience sample of seven cargo ships, at four time points with about 3 months in between.	127 seafarers	Job Demands-Resources	The degree of fatigue varies depending on an individual’s position on the ship. Irregular working hours continue to disrupt the circadian rhythm, leading to higher levels of fatigue. Fatigue is influenced by the ship’s schedule, especially the number of port stops.
Shan and Neis (36)	North America (Canada)	Qualitative study	Interviews	11 seafarers, 4 offshore managers and 2 key information providers from authority.	Job Demands-Resources	The more times a port is visited (and to a certain extent, the fewer days spent sailing at sea), the higher the level of fatigue. In contrast, night duty and the number of days spent in port have no effect on fatigue. Supportive social interaction can alleviate fatigue (main effect), but when the number of port visits increases, its effect weakens (interaction effect).
Zhao et al. (33, 34)	Asia (China)	Mixed methods (quantitative study and qualitative study)	Quantitative questionnaire surveys and qualitative interviews	924 seafarers and 12 managers	Cardiff Seafarers’ Fatigue Research Programme and online interviews	During the epidemic period and after it, the risk factors for fatigue are different. During the epidemic, the risk factors are often related to the epidemic restrictions. After the epidemic, the risk factors are more likely to be associated with the company’s fatigue risk management policies and practices.
Zhao et al. (33, 34)	Asia (China)	Mixed methods (quantitative study and qualitative study)	Interviews	776 seafarers and 11 managers	Cardiff Seafarers’ Fatigue Research Programme and online interviews	The fear of infection, the increase in workload, the deprivation of coastal leaves, and the extension of service time all contributed to physical and mental exhaustion.
Dohrmann et al. (23)	Europe (Denmark)	Cross-sectional study	Self-administered questionnaires collected within 5 months	193 seafarers	The Swedish Occupational Fatigue Inventory and the Copenhagen Psychosocial Questionnaire	The four out of the five sub-dimensions of work–family conflict and fatigue were found to be positively correlated: lack of energy, physical discomfort, lack of motivation, and sleepiness, while more supervisor support was associated with less lack of energy, physical exhaustion and lack of motivation. Moreover, supervisor support was regarded as the influence on the physical sub-dimensions of mild work–family conflict-related fatigue.
Rajapakse and Emad (39)	Oceania (Australia)	Qualitative study	The respondents conducted one-on-one interviews with seafarers at different stages of their careers.	63 seafarers	Offline interviews	Four categories of maritime fatigue among seafarers that led to accidents were identified. These were inadequate staffing, unstable work conditions, insufficient rest regulations at work, and technological advancements.
Yancheshmeh et al. (22)	Europe (the United Kingdom)	Cross-sectional study and cognitive behavioral tests	Self-administered questionnaires were collected at a certain stage during the day. And neurobehavioral assessment tests are conducted in a quiet room.	70 seafarers	Psychomotor Vigilance Task (PVT) performance, Arrow Flanker Task performance, the Pittsburgh Sleep Quality Index (PSQI), and the Karolinska Sleepiness Scale (KSS)	Shift work that contradicts circadian rhythms, particularly during peak sleep pressure periods, induces short-term cognitive deficits and acute fatigue. Long-term consequences include chronic fatigue and sleep deprivation. Aging deteriorates sleep quality and exacerbates fatigue. Increased maritime experience and heightened onboard responsibilities further elevate crew fatigue levels. Daytime sleeping notably diminishes sleep quality for officers and is insufficient for full recovery, contributing to their fatigue.
Andrei et al. (37)	Oceania (Australia)	Cross-sectional study	Survey packages with prepaid return envelopes were distributed to employees working on the company’s vessels.	199 seafarers	The Questionnaire on the Experience and Assessment of Work	Working under time pressure and the need for alertness has a different relationship with chronic fatigue, and the need for alertness shows a stronger correlation. Work autonomy also shows a direct negative correlation with chronic fatigue.
Hystad et al. (26)	Europe (Norway)	Cross-sectional study	Self-administered questionnaire divided into two parts, was distributed at two different time points, with an interval of approximately one week.	151 seafarers	Organizational-level safety climate scale, the Swedish Occupational Fatigue Inventory and the Pittsburgh Sleep Quality Index	Sleep quality, safe climate, and two covariates jointly explained 32% of the variance in fatigue. Fatigue is related to poor sleep quality. Safe climate can predict sleep quality and fatigue.
Pauksztat et al. (27)	Europe (Sweden)	Cross-sectional study	Self-administered questionnaires through an online survey	622 seafarers	PHQ-4 scale and the seafarers’ fatigue scale	There is a significant negative correlation between the support from fellow crew members and mental health problems, as well as between the support from fellow crew members and fatigue. This indicates that the support from fellow crew members reduces mental health problems and fatigue. The direct impact of network quality on mental health problems and fatigue is not significant. The impact of the COVID-19 pandemic has increased chronic fatigue.
Yang et al. (28, 29)	Asia (China)	Cross-sectional study and body data collection	Questionnaires before the formal test. Wearable ECG and EEG equipment Devices are used to collect the physiological data.	24 seafarers	Perceived Stress Questionnaire and Safety Behavior Questionnaire	Age and health affect seafarer fatigue. Older, healthy seafarers are less prone to fatigue, while long working hours and short departure intervals increase fatigue. Traffic complexity and monotonous environments significantly impact physical fatigue and alertness. Perceived stress and safety behaviors also influence fatigue, with safety behaviors having a notable effect on crew alertness and fatigue states.
Cham et al. (38)	Oceania (Australia)	Cross-sectional study	Self-administered questionnaires	1,026 seafarers	The Questionnaire on the Experience and Evaluation of Work and Occupational Fatigue Exhaustion Recovery (OFER) measure	Overload and underload significantly affect chronic fatigue and mental health. Insufficient workload frequency in seafarers’ work predicts higher chronic fatigue levels. Despite no hypotheses being proposed, insufficient workload is more strongly associated with chronic fatigue and mental health than excessive workload.
Dohrmann et al. (25)	Europe (Denmark)	Cross-sectional study	Self-administered questionnaires	193 seafarers	The Swedish Occupational Fatigue Inventory and the Copenhagen Psychosocial Questionnaire	Effects tended to be stronger for psychological than for physical aspects of fatigue and adding sleep satisfaction improved predictive power while reducing the effect sizes for job demands and control, suggesting that parts of the effects of perceived job demands and control are mediated via sleep quality.
Mansyur et al. (32)	Asia (Indonesia)	Cross-sectional study	Self-administered questionnaires	127 seafarers	The Pittsburgh Sleep Quality Index (PSQI) and Work-Family Conflict Scale (WCFS)	The decisive factors for the subjects classified as being fatigued were long working hours (> 72 h per week), poor sleep quality, and work–family conflict. However, personal and occupational factors, including age, marital status, duration on board, length of seafaring experience, smoking status, and coffee and alcohol consumption, were not significantly associated with crewmember fatigue.
Bal et al. (31)	Asia (Turkey)	Cross-sectional study	Self-administered questionnaires and Lactate Test	31 seafarers	The Analytic Hierarchy Process (AHP) method	Sleep has a substantial role in the occurrence of fatigue in seafarers. Seafarers are generally highly fatigued, and the fatigue levels increase particularly during port calls.
Hystad and Eid (74)	Europe (Norway)	Cross-sectional study	Self-administered questionnaires returned in the form of an anonymous sealed envelope	742 seafarers	The Swedish Occupational Fatigue Inventory	Psychological capital is a valuable positive resource for seafarers, which can help them cope with work in isolated and restricted environments. Psychological capital significantly negatively predicts fatigue and can buffer the negative impact of working hours at sea on fatigue. The influence of environmental stress (such as noise and ship movement) on fatigue is weakened in individuals with high psychological capital.

### 3.2 General characteristics of included studies

The general characteristics of 18 studies are demonstrated in Table 1. The number of participants in the studies ranged from 24 to 1,026. Eight studies were conducted in Europe (22–29, 74), Six in Asia (30–35), one in North America (36) and three in Oceania (37–39). Two studies were qualitative (36, 39), three were mixed methods (33–35), and the rest were quantitative.

As to methods utilized for assessing fatigue, one study used professional equipment to test Psychomotor Vigilance Task (PVT) performance and Arrow Flanker Task performance (22) of seafarers, one study used wearable ECG and EEG equipment devices (28, 29) while conducting a field study, and one study used the Lactate Test (31). Three mixed-methods studies applied interviews with seafarers and managers (33–35). And one qualitative study applied interviews with seafarers, managers, and officers from the authority (36).

The number of subjects of quantitative studies ranged from 24 to 1,026. The participants of five studies comprised female seafarers (23, 24, 30, 37, 38).

## 4 Factors affecting seafarers’ fatigue

### 4.1 Individual factors

#### 4.1.1 Physiological factors

Four studies included age in their research scope (22, 25, 28, 29, 32). The age of seafarers has no significant impact on their fatigue levels (25, 32), but as people get older, the fatigue levels of seafarers have significantly decreased in the United Kingdom and China (22, 28, 29). Seafarers with poorer health conditions are more prone to fatigue (28, 29).

Sleep deprivation, disruption, and poor quality are the primary causes of fatigue among seafarers. This shows that the four-hour shift system for seafarers disrupts their sleep patterns and exacerbates circadian rhythm disorders (31). At the same time, poor sleep quality significantly increases the risk of fatigue (22, 26, 32–35). The sleep quality of crew members is influenced by various factors, including shift schedules (such as night duty), ship noise, and rocking, etc. Environmental stress indirectly exacerbates fatigue by interfering with sleep, especially in the operation of roll-on/roll-off ships with frequent night berths (74). Seafarers with poor sleep quality exhibit higher reaction times, more lapses of attention, and poorer selective attention control during cargo handling operations (22). Poor sleep quality leads to insufficient relief of acute fatigue, which accumulates into chronic fatigue. Sleep satisfaction is significantly correlated with all dimensions of fatigue, and the two show a negative correlation. Sleep satisfaction plays a partial mediating role between work stress (work demands and work control) and fatigue; that is, work stress may affect fatigue levels by influencing sleep quality (25). Moreover, prolonged use of digital devices disrupts sleep patterns, leading to loss of attention and fatigue (30).

#### 4.1.2 Psychological factors

The level of stress is positively correlated with the level of fatigue, and the level of safety behavior ability is negatively correlated with fatigue (28, 29). Seafarers’ time pressure (such as heavy workload and the need to complete tasks quickly) is related to chronic fatigue, which mainly affects them indirectly through sleep problems and incomplete recovery, not directly causing acute fatigue. The demand for alertness (such as long duty hours and monitoring tasks) has a more significant impact on chronic fatigue, both directly and indirectly, and has a prominent negative impact on sleep quality. Meanwhile, work autonomy has a buffering effect on chronic fatigue under high time pressure, while it alleviates sleep problems under low to medium alertness demands (37).

Work–family conflict is significantly associated with the four dimensions of fatigue (lack of energy, physical discomfort, lack of motivation, and drowsiness). This indicates that when employees feel conflicts between their work and family roles, they are more likely to feel fatigued. The impact of work–family conflict on psychological fatigue (such as lack of energy and lack of motivation) is greater than that on physical fatigue (such as physical discomfort and physical exhaustion) (25).

Psychological capital (self-efficacy, optimism, hope, resilience) is the strongest variable for predicting fatigue, and it has a significant negative correlation with fatigue in both supply ships and roll-on/roll-off ships, buffering the negative effects of long voyages (74).

#### 4.1.3 Sociological factors

The unmarried seafarers in the Turkish sample showed significantly higher fatigue levels (31). However, a study has shown that marital status is not significantly associated with seafarers’ fatigue (32). Seafarers working in the deck department and those at higher ranks (such as captains and senior crew members) reported higher levels of fatigue (24, 33, 34). Seafaring experience is weakly negatively correlated with fatigue among supply ships and tugboats, but the practical significance is limited, indicating that experience has a non-significant effect on the alleviation of fatigue (32, 74). While in the Chinese sample and the roll-on-roll-off ship sample in Norway (33, 34, 74), it has no significant direct effect on fatigue. But it may indirectly affect the level of fatigue through interactions with other factors, such as work stress, sleep quality, environmental adaptation, etc. (33, 34).

In addition, long-distance commuting, the direct connection between commuting and work, as well as the complexity of commuting patterns, are all factors that affect seafarers’ fatigue (36).

### 4.2 Work environment

Safety climate is negatively associated with both fatigue and sleep quality. The better the safety climate is, the lower the fatigue level of seafarers will be. More positive appraisals of the safety climate were related to decreases in reported fatigue and poor sleep quality (26).

External support (such as support from the company, family, friends and shore-based organizations) significantly reduces the fatigue of seafarers (27). Supportive social interactions on board can significantly lower the fatigue level (24, 27). There is a significant negative correlation between superior support and fatigue (25).

Work demands (39), such as the number of port stops (24), long working hours and high workload (22, 31, 35, 36) have a significant impact on the fatigue of crew members, especially the psychological fatigue dimension (25). Crew members working at night show higher levels of drowsiness and poorer cognitive performance than those working during the day (22).

Environmental factors such as noise, ship rolling, frequent port calls, and inappropriate temperature/lighting in the sleeping quarters directly lead to an increase in fatigue levels (74). The movement of the ship in rough seas and noise disturbances affect the sleep quality of seafarers (35), even with rest time, they may not be able to effectively recover their physical strength (36). The frequency of port calls, congestion in the shipping lanes, and the threat of piracy/terrorism (requiring the implementation of the ISPS protocol) are among the environmental factors that have the most significant impact on seafarers’ fatigue (31).

### 4.3 Management

Insufficient crew allocation, with the number of crew on board falling below the minimum standard required for safe operation, leads to an increase in each crew member’s workload, thereby causing fatigue (39).

The existing work-rest regulations fail to adequately consider the rest needs of seafarers in dynamic maritime environments. The current regulations are incomplete and ambiguous, leading to fatigue problems (39). Post-pandemic policy and regulation updates have also contributed to seafarers’ fatigue (33, 34).

Technological advancements also have an impact. Although technological progress has improved work efficiency, it has failed to alleviate the workload of seafarers. The use of non-standard equipment increases the difficulty for seafarers, their reliance on automation technology, and the lack of human-centered design in technological advancements lead to inconvenient equipment usage, all of which may contribute to more fatigue (39).

Navigation plans, including tight port arrival schedules and route planning for adverse weather conditions, exacerbate the pressure, thereby leading to increased fatigue among seafarers (31).

Fatigue risk management systems (including fatigue training, work time policies, and the effectiveness of fatigue risk management systems) have a significant effect in alleviating fatigue (33, 34). The management practices of shipping companies and their commitment to fatigue alleviation have a significant impact on seafarers’ fatigue levels. For example, compared to Chinese companies, the fatigue management level of European shipping companies is higher, and the fatigue levels of European seafarers are lower than those of Chinese seafarers (35).

### 4.4 Interactions

High-intensity work schedules have been shown to induce sleep deprivation (31), while shift systems contribute to sleep fragmentation (36). Similarly, rigorous work arrangements mitigate the impact of night shifts and sea days on fatigue (24). Extreme environments, such as polar navigation, disrupt sleep through noise and motion while increasing psychological stress (31, 36), exacerbating sleep disorders (24, 37). Additional work further compresses sleep time (24).

**Figure 2:**
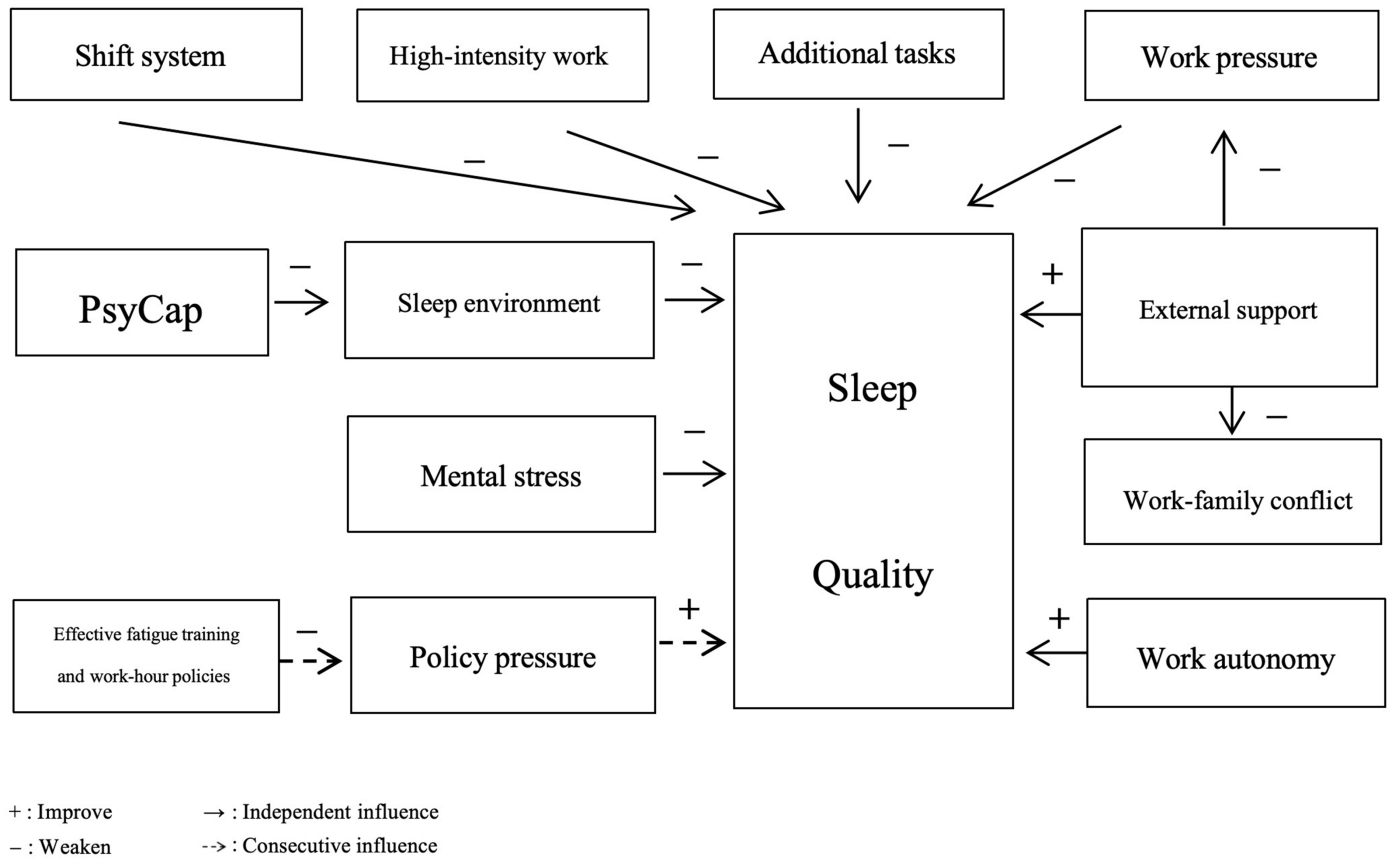
A summary diagram of the interactions among various influencing factors.

Captains experience poorer sleep quality due to heightened work pressure (22, 25). Psychological capital can alleviate the effects of environmental stress on fatigue (74). Peer support counteracts the negative impact of work stress on fatigue (24). Social support also interacts with time pressure, buffering the adverse effects of high time pressure on chronic fatigue and incomplete recovery (37). At low to moderate levels of vigilance demand, work autonomy and social support significantly reduce sleep problems, though these effects are not significant under high vigilance demand (37). Supervisory support can buffer the negative impact of work–family conflict on fatigue (23). Effective fatigue training and work-hour policies can partially offset the negative impacts of pandemic policies and inspection pressures, improving sleep quality (33, 34). These relationships are illustrated in Figure 2.

## 5 Discussion

This review uses the PRISMA method to analyze the relevant literature and systematically describes the research results on the factors influencing seafarers’ fatigue over the past decade. The review includes a total of 18 studies.

Compared with other reviews in this field (20), this review incorporates more studies that involve female seafarers as the subjects, with 38.9% of the studies having subjects from two or more nationalities. In terms of sample selection, it is more comprehensive and integrated. In terms of research methods, more of the evaluated studies used quantitative research, while a small portion employed mixed research methods or pure qualitative research. In the studies analyzed in this review, the data collection methods included online or offline interviews, online questionnaires, offline mailed questionnaires, and the use of professional physiological data collection equipment, and most were conducted in the form of seafarers’ self-reports. Almost all the quantitative studies in this review are mentioned later, and the self-reporting form may have certain subjectivity due to some other social and psychological reasons and cannot fully objectively reflect the facts.

This review once again demonstrates the extremely high prevalence of fatigue among seafarers, despite the implementation of work and rest time regulations as well as relevant industry norms. Fatigue remains a pressing issue in the shipping industry (40). The results of the review reveal the three main factors influencing seafarers’ fatigue, which are the seafarers’ personal factors, work environment, and management.

Among the individual factors of seafarers, they can be classified as physiological factors, psychological factors, and sociological factors. In numerous studies, sleep has been identified as the most significant factor affecting fatigue. Moreover, as a direct factor influencing fatigue, sleep, along with other environmental factors such as noise, ship movement, frequent port calls, discomfort in sleep cabins regarding temperature/lighting, and work patterns, also indirectly affects the fatigue of seafarers (74). In studies from different countries, there are varying results regarding whether age, marital status, and sailing experience can affect the fatigue of seafarers (22, 25, 28, 29, 31–34, 74). The main reason for this might be sociocultural variables in this review. As different sociocultural factors have a profound impact on human behavior and social structure. These factors include social norms, cultural values, language, education, religious beliefs, socioeconomic status and ethnic background etc. (41). For instance, the extent to which religion influences marriage might explain the contradictory findings in this study regarding marriage. The marriages in Indonesia and Turkey are both influenced by Islamic law (42). Indonesian marriage law reflects Islamic principles to a certain extent but is also affected by local customs (43). While in Turkey, a large-scale legal reform was carried out in the early 20th century, which to some extent reduced the direct influence of religion on marriage (44).

This review also found that work autonomy and psychological capital, as the psychological factors of seafarers themselves, can effectively alleviate seafarers’ fatigue. Psychological capital is regarded as an important internal resource that can help individuals cope with challenges and pressures at work, thereby reducing fatigue (45). This fully demonstrates that when the inevitable feeling of fatigue occurs, seafarers can alleviate their fatigue by mobilizing their subjective initiative in the form of regaining control over life and work.

Among the work environment factors affecting seafarers’ fatigue, soft environmental factors, such as a safety climate, external support, work demands, and work patterns, are more frequently mentioned. That is, good interpersonal and workplace relationships play an important role in alleviating seafarers’ fatigue. And these factors are easily associated with another major psychological problem faced by seafarers, which is seafarers’ loneliness. The improvement of these factors can also significantly alleviate seafarers’ loneliness. Perhaps the two psychological states have a certain symbiotic and correlational relationship. Currently, no scholar has conducted a correlation study on the two. At the same time, reasonable and non-overloaded work arrangements also play an important role in improving seafarers’ fatigue status.

Regarding management factors, on the one hand, there are deficiencies in the regulations within a country’s shipping industry. However, it should be clear that inadequate regulations are one issue, while poor enforcement and supervision of regulations are a more serious problem. According to a report from World Maritime University conducted on 6,304 seafarers from 113 countries, 64.3% of the respondents reported adjusting their work/rest records. And nearly half of the respondents questioned the effectiveness of the current regulations in addressing fatigue (46). Seafarers are reluctant to report fatigue issues due to concerns about negative evaluation reports and may even fabricate rest records (39). The “falsification” of seafarers’ work/rest records has been reported over decades (46, 47). This indicates a systematic failure in the implementation and supervision of the international maritime regulatory framework.

On the other hand, the other two management factors that cause seafarers fatigue are insufficient manning and the use of various new technologies on board. The report from the World Maritime University also confirmed this view, stating that 87.6% respondents believed there was an imbalance between the available crew and the required workload, and 65.8% respondents thought that the implementation of new technology has not alleviated the workload (46). The management’s need to cut costs might be able to explain these.

Further, this involves more technical issues, such as the ways to properly deal with digital fatigue and technostress in the context of technological advancement. The shipping industry’s use of digital technology often stops at operational improvements rather than driving full digital transformation, missing its full potential (48). Maritime technostress, digital overload, and change resistance should be focused on, as there is an industry-wide underestimation of technostress’s effects, risking the wellbeing of employees amid digital shifts (49).

Moreover, the management cultures of different countries, including the degree of employee care, the role of trade unions in representing workers’ interests, and the management layer’s preference for centralized or participatory decision-making methods, all have certain impacts on crew fatigue.

Regarding the interrelationships among various risk factors, they can be explained through physiological and psychological mechanisms, respectively. From a physiological perspective, shift work, especially night shifts, disrupts the body’s natural circadian rhythm (50). The circadian rhythm, an internal biological clock regulating the sleep - wake cycle, is influenced by external factors such as light exposure (51). Irregular light exposure and inconsistent sleep times caused by shift work can lead to a misalignment between the circadian rhythm and the external environment, thereby affecting sleep quality (52). Research indicates that shift workers often struggle to adapt to night-time work and daytime sleep, resulting in reduced sleep duration, fragmented sleep, and decreased sleep efficiency (53). The unique light conditions in polar environments, such as the midnight sun and polar night, also severely disrupt the human circadian rhythm (54). Moreover, the uncertainty and potential hazards in extreme environments can activate the body’s stress response system, leading to an increased secretion of stress hormones such as cortisol (55). These hormones interfere with sleep, making it difficult for individuals to enter deep sleep and reducing sleep quality (56).

From a psychological mechanism perspective, psychological stress can lead to cognitive arousal in seafarers, meaning that their brains remain in a state of high alert and are unable to relax and fall asleep (57). Work-related stress, such as excessive workload and tense interpersonal relationships, can trigger worry and anxiety, making it difficult for people to fall asleep or maintain a sleep state (58). This effectively explains why seafarers’ sleep quality declines due to stress-related issues. Research has found that there is a significant negative correlation between work pressure and sleep quality (59), which also supports the findings in the study. Perhaps because effective fatigue training and work time policies can alleviate the stress of seafarers, thereby partially offsetting the negative impacts brought about by pandemic policies and inspection stress. Peer support and social support can enable seafarers to better control their work progress and working methods, enhancing their autonomy in work. All of these contribute to reducing work pressure and improving sleep quality. As for psychological capital, it helps individuals to conduct cognitive reappraisal, that is, to change their perception of stress and view it as a challenge rather than a threat (60). This positive cognitive approach can lower the level of cognitive arousal and promote relaxation and sleep for seafarers. Psychological capital is related to better emotional management skills (61). Seafarers with higher psychological capital may be better at regulating their emotions, reducing the interference of negative emotions such as anxiety and depression on sleep. Moreover, work autonomy enables employees to better balance work and life, thereby reducing sleep problems caused by work occupying time from life (28, 29). The resource conservation theory suggests that individuals tend to protect and accumulate resources. When resources are threatened, individuals will feel stressed and fatigued (62, 63). Here, superior support can be regarded as an important resource that helps seafarers cope with resource depletion caused by work–family conflicts, thereby reducing fatigue.

### 5.1 Beneficial approaches to seafarers’ fatigue

The reviewed studies, through the diversity of their samples (such as the nationality of the crew, the shipping company or the flag state, gender), indicate that fatigue is a widespread problem in international maritime transportation worldwide and cannot be attributed to specific regions or stakeholders. The improvement plans provided by each study at the end of the article also converge in essence, mainly focusing on the aspects for improvement.

In conclusion, the solutions these studies provide can be summarized into five points. First, improve working conditions, provide a comfortable sleeping environment, and reduce interference factors such as noise and vibration. Second, arrange working hours reasonably, and flexibly schedule working hours. Third, provide more psychological support, through training and psychological counseling, to help seafarers cope with work pressure and fatigue. Fourth, strengthen safety culture construction and enhance seafarers’ safety awareness and behavior. Fifth, improve the corresponding management system, including the rest and training regulations, technical equipment, and risk management. According to a report from World Maritime University, workload, long working hours, and sleep deprivation are the top three fatigue risk factors (46). These solutions covered the most critical issues mentioned in the report. However, they lack the solutions to address the insufficient manning, which more seafareres thought would be the highest priority long-term solution for managing fatigue (46).

Therefore, it is necessary to formulate solutions that involve all parts of the entire industry, including seafarers, managers, and maritime authorities, to systematically manage seafarers’ fatigue. Luckily, the application of artificial intelligence (AI) technology in ensuring driver safety and preventing fatigue-related accidents has witnessed significant progress. It holds great potential for revolutionizing the current driver fatigue monitoring systems (64). Advanced AI technology may assist relevant entities in developing more reliable and efficient solutions for seafarers’ fatigue management. Beyond the seafarers’ fatigue risk management system, improvements or the formulation of more humane rules are needed. Especially, IMO has reached a preliminary framework for the carbon tax-related issues of the international shipping industry through MEPC 83 in 2025, and the “net zero” goal is being firmly implemented (65, 66). This undoubtedly will further affect various aspects of the seafarers’ profession, including technical training, employee wellbeing, work demands, etc., all of which will affect the seafarers’ fatigue.

Of course, such a process may be quite challenging, because the specific employment conditions of seafarers (temporary employment, responsibility of the seafarer agency) may prevent them from fully expressing their interests, thus making it impossible for them to participate actively and effectively in it; in addition, there is also a rather serious segmentation phenomenon in the labor market of the international shipping industry (67).

### 5.2 Strengths and limitations

This scoping review follows the framework of Arksey and O’Malley (21), the PRISMA-ScR flowchart, and the established inclusion criteria. Since there are no restrictions on the types of studies, various research designs are included, such as quantitative, qualitative, or mixed methods. The source countries of the studies and the diversity of the included samples are relatively high, making this review representative to some extent. And this review examines and summarizes the interactive relationships among various risk factors as presented in the studies.

Regarding the limitations of the review, first, the quantitative studies included all used self-reports, which could potentially lead to self-report bias. Second, the correctness of the analysis of objective parameter measurement results in the studies needs to be verified. Third, the studies reviewed vary widely in ship types, crew roles, routes, and cultural contexts. While diversity is a strength, it also weakens generalizability. Further, this review misses an important risk factor, which is the lack of skills among seafarers. If seafarers lack certain specific skills, especially those related to digital transformation, it may lead to increased work pressure and subsequently cause fatigue (68, 69).

## 6 Conclusion

This review indicates that the issue of fatigue among seafarers has always existed. The factors influencing seafarers’ fatigue can be classified as factors related to the seafarers themselves, factors related to the working environment, and management factors. These results comprehensively provide solutions for addressing seafarers’ fatigue at both the individual and organizational levels. At the individual level, focusing on the accumulation of psychological capital and enhancing work autonomy can alleviate fatigue. More solutions need to be implemented at the organizational level, including providing a better sleep environment, providing more external support, flexibly handling work demands and work patterns, and improving technology and management measures. It is also noticed that some studies have reached opposite conclusions. Further, as external support served as an impactful factor, seafarers’ fatigue may have a deeper connection with seafarers’ loneliness. In all, some situations allow for more detailed research. These require scholars to design more scientific experiments and conduct further research.

## Data Availability

The original contributions presented in the study are included in the article/supplementary material, further inquiries can be directed to the corresponding author.
